# Modelling Deep Water Habitats to Develop a Spatially Explicit, Fine Scale Understanding of the Distribution of the Western Rock Lobster, *Panulirus cygnus*

**DOI:** 10.1371/journal.pone.0034476

**Published:** 2012-04-10

**Authors:** Renae K. Hovey, Kimberly P. Van Niel, Lynda M. Bellchambers, Matthew B. Pember

**Affiliations:** 1 The UWA Oceans Institute and School of Earth and Environment, Faculty of Natural and Agricultural Sciences, The University of Western Australia, Perth, Western Australia, Australia; 2 Western Australian Fisheries and Marine Research Laboratories, Department of Fisheries, Government of Western Australia, Perth, Western Australia, Australia; National Institute of Water & Atmospheric Research, New Zealand

## Abstract

**Background:**

The western rock lobster, *Panulirus cygnus*, is endemic to Western Australia and supports substantial commercial and recreational fisheries. Due to and its wide distribution and the commercial and recreational importance of the species a key component of managing western rock lobster is understanding the ecological processes and interactions that may influence lobster abundance and distribution. Using terrain analyses and distribution models of substrate and benthic biota, we assess the physical drivers that influence the distribution of lobsters at a key fishery site.

**Methods and Findings:**

Using data collected from hydroacoustic and towed video surveys, 20 variables (including geophysical, substrate and biota variables) were developed to predict the distributions of substrate type (three classes of reef, rhodoliths and sand) and dominant biota (kelp, sessile invertebrates and macroalgae) within a 40 km^2^ area about 30 km off the west Australian coast. Lobster presence/absence data were collected within this area using georeferenced pots. These datasets were used to develop a classification tree model for predicting the distribution of the western rock lobster. Interestingly, kelp and reef were not selected as predictors. Instead, the model selected geophysical and geomorphic scalar variables, which emphasise a mix of terrain within limited distances. The model of lobster presence had an adjusted D^2^ of 64 and an 80% correct classification.

**Conclusions:**

Species distribution models indicate that juxtaposition in fine scale terrain is most important to the western rock lobster. While key features like kelp and reef may be important to lobster distribution at a broad scale, it is the fine scale features in terrain that are likely to define its ecological niche. Determining the most appropriate landscape configuration and scale will be essential to refining niche habitats and will aid in selecting appropriate sites for protecting critical lobster habitats.

## Introduction

Marine ecosystems worldwide are under increasing pressure due to anthropogenic impacts such as coastal development, pollution and overfishing [1]–[5]. Previous studies have suggested that the impacts of these pressures are being reflected in a decline in biodiversity, outbreaks of marine pests and declining habitat quality [3]–[5]. In recent years ecosystem-based fisheries management (EBFM) has been implemented in an attempt to manage exploited marine resources in a more holistic manner [6]–[9]. One component of EMFB is marine spatial management such as multiple use areas or reserves [6]. However, the implementation of any type of spatial management of exploited species requires an understanding the interaction of the organism with their physical habitat [10], [11]. Establishing key environmental characteristics that influence the distribution of fish and crustacean populations is thus a critical aspect of maintaining fisheries resources [12]–[14].

The western rock lobster *Panulirus cygnus* George is endemic to the west coast of Australia, with a range from the North West Cape south to Cape Leeuwin [15], where it is the dominant benthic consumer. It also supports Australia's most valuable single-species fishery and significant recreational fisheries [16]. A dramatic decline in puerulus numbers in 2008/09 [16] resulted in management actions to reduce catch to ensure sustainability raised questions about the influence of ecological interactions and processes in determining lobster abundance and distribution. The need to understand interactions and processes that may potentially drive the abundance and spatial distribution of rock lobster resulted in the establishment of a closed area in the deep water (>40 m) portion for the fishery at Leeman in 2011.

The life history of the western rock lobster is spatially segregated; juveniles dominate inshore reefs and majority of sexually mature adults are found on offshore reefs [15], [17]. Most western rock lobsters are targeted when they migrate offshore as they approach sexual maturity and reach legal minimum size [15]. Despite the importance of offshore reefs and deepwater habitats to the sustainability of the species, little is known about what factors of the environment influence their distribution, beyond the very generic requirements of reef and mixed assemblages dominated by the kelp *Ecklonia sp*. [18].

With very little known about deepwater lobster habitats, species distribution models offer (1) the potential to develop detailed baseline information of the spatial ecology of this particularly cryptic species, and (2) the chance to better understand how environmental characteristics affect species distribution patterns. Both outcomes have the potential to provide useful spatially-explicit information on critical habitats that can be incorporated into fisheries management. Originally applied to terrestrial ecology [19], species distribution modelling is now widely used in marine research [20]–[22]. Using full coverage bathymetry and tow video data, species distribution models can be used to map terrain, substrate and seafloor biota, providing detailed information on the variation, composition and configuration of seafloor habitats. Predictive species modelling can then proceed by developing statistical or mathematical models that link the target species to observed or measured habitat attributes, leading to predictions of contemporary species distributions. For example, Holmes et al. [27] conducted broad scale marine mapping of sessile benthos at Point Addis Marine Park, Australia, using terrain analysis and species distributions models of biota (macroalgae, rhodoliths, sponges, ascidians, and soft corals). Chatfield et al. [13] used species distribution models and data on fish and habitats from baited underwater video systems (BRUVS) to assess the level of influence of substrate type, macroalgal type and sessile biota on the distribution of fishes. Work on crustaceans has included the development of habitat maps and catch data for the spiny lobster (*Panulirus argus*) to improve stock assessment [23]. Also, Galparsoro et al. [12] used ecological niche factor analysis for developing species distribution models, and found the distribution of the European lobster (*Homarus gammarus*) was mainly influenced by distance to rock substrates, benthic position index, wave flux at the seafloor, and bathymetry.

In this study, species distribution modelling was based on lobster pot data from the closed area at Leeman. Classification trees (CTs) were used to define species distribution, because they are well suited to exploring and modelling complex ecological data [20], [24], [25]. They also provide greater deviance explained compared to general additive models (GAMs), while maintaining adequate predictive performance [26]. A necessary component to species distribution is high resolution, spatially explicit, continuous data such as detailed habitat maps and bathymetry, a very rare commodity in marine deep water environments [27]. Thus, habitat maps of the terrain, substrates, and biota at the study area were created first, derived from hydroacoustic surveys and towed video. These three data sets were then used to develop CT models for predicting the distribution of the western rock lobster.

## Methods

### Study area

**Figure 1:**
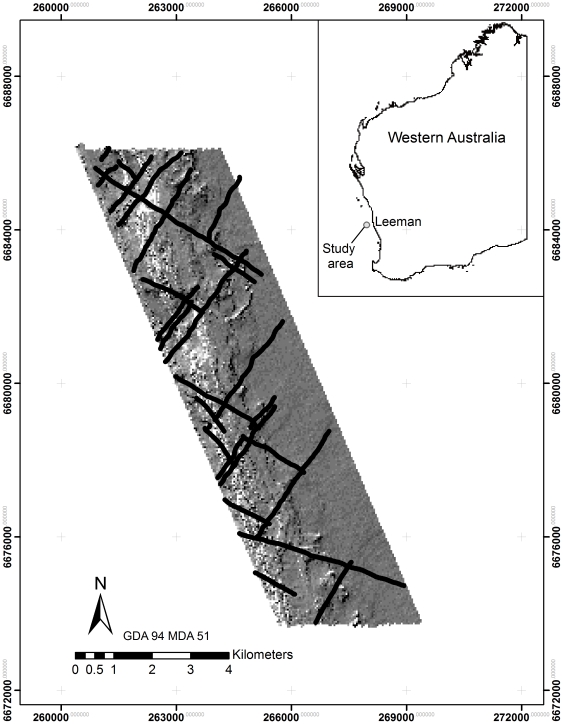
Study location (top right) and map of bathymetry from the hydroacoustic survey used for planning locations of video observations (black lines).

The study site was located approximately 30 km offshore from Leeman (29°56.94 S, 114°58.74E), Western Australia (Fig. 1). A portion of the study site was closed to lobster fishing in March 2011 to provide a research area in the absence of lobster exploitation.

### Lobster sampling and data collection

Lobsters were sampled in water depths 45–80 m using standard baited commercial pots, with closed escape gaps, deployed from chartered commercial western rock lobster vessels as part of the western rock lobster independent breeding stock survey (IBSS). This annual standardised ten day fishery independent survey has been conducted in mid to late October at a number of sites along the Western Australian coast since the early 1990s [28]. The area off Leeman area was added to the existing survey in 2008, providing three years of pre-closure information on the distribution, size and abundance of lobsters. Three hundred and thirty (330) standard commercial pots were deployed, 400 m apart, in 11 lines of 30 pots running north to south parallel with the coast. Each pot has its own GPS coordinate so the pot locations are consistently sampled between years. The data collected include the number of lobsters per pot, carapace length in millimetres, sex, reproductive state and general condition of lobsters caught in each pot. All lobsters were released following data collection. For this study, we focus only on the presence or absence of lobsters in pots.

### Bathymetry and biological data collection

To complete full coverage information on terrain, substrates and benthic biota, detailed information of the seafloor was captured. A full coverage dataset for the study area consisted of bathymetry from hydroacoustic surveys collected in 2010, using a SEABAT 8101 Reson Multibeam. Bathymetry spot heights were averaged using the moving window technique to produce a digital bathymetry model at 3×3 m resolution. A detrended bathymetry was created to remove depth gradient effects. A trend was calculated from the bathymetry data points (at the same resolution as the bathymetric data set: 3 m) using a linear polynomial. The trend was then subtracted from the bathymetry to create the detrended surface. This enabled the development of important variables such as seafloor rugosity, topographic position, reef relief and aspect, which would otherwise be affected by the strong depth gradient trend in the bathymetric data. Secondary datasets assessing geomorphic terrain features were developed from the bathymetry and detrended bathymetry (Table 1).

**Table 1:** Description of the datasets derived from bathymetry used as predictors of substrate and biota (adapted from Holmes et al. [27]).

Predictor datasets	Definition	Predictor Codes
Bathymetry	Depth relative to the Australian Height Datum	DTH
Bathymetry (detrended)	Bathymetry with the depth gradient removed. A trend is calculated from the bathymetry data points (at the same resolution as the bathymetric data set: 3 m) using a linear polynomial. The trend is then subtracted from the bathymetry to create the detrended surface.	DETRND
Aspect	Direction of the steepest slope (0–360°), calculated on 3×3 pixel area	ASP
Slope	Average change in elevation with distance calculated on 3×3 pixel area	SLP
Profile curvature	Measure of concave/convexity parallel to the slope (e.g., hill cross-section), calculated on 3×3 pixel area	PROCURVDT
Plan curvature	Measure of concave/convexity perpendicular to the slope (e.g., contour lines), calculated on 3×3 pixel area	PLANCURV
Focal analysis	Statistical operation that computes a value for each cell as a function of cells that are in a specified neighborhood around a focal cell, calculated as standard deviation of surface area with kernel radius of 7 m and 21 m.	F7S (stdev, 7 m) F21S (stdev, 21 m) F21M (mean, 21 m)
Curvature	Combined index of profile and plan curvature	CURV
Hypsometric index	Indicator of whether a cell is a local high or low point within a neighborhood of 12.5, 25 and 62.5 m kernel radius	HYP5 (12.5 m) HYP10 (25 m) HYP25 (62.5 m)
Range (local relief)	Maximum minus the minimum depth in the local neighborhood of 12.5, 25 and 62.5 m kernel radius	RNG 5 (12.5 m) RNG 10 (25 m) RNG 25 (62.5 m)
Standard deviation	Standard deviation of depth within a neighborhood of 12.5, 25 and 62.5 m kernel radius	STD 5 (12.5 m) STD 10 (25 m) STD 25 (62.5 m)
Rugosity (surface area)	Actual surface area of local neighborhood	SURFA

Substrates and benthic biota were observed using video footage from an underwater camera towed behind a boat travelling 1–2 knots per hour. The camera was held at approximately 1 m above the seafloor and the position georeferenced using an Ultra Short Base Line (USBL) acoustic positioning system linked to a GPS with satellite differential correction. Video footage was archived with digitally superimposed GPS time stamps at 1 second intervals. Sampling was designed to ensure a broad geographic coverage of the study area with sufficient numbers of georeferenced data for modelling and mapping. Seafloor information from the hydroacoustic survey enabled a design that captured benthos across the full range of habitats (Fig. 1). A total of 55 km of seafloor was sampled with the video over a 52 km^2^, giving a ratio of video footage to area of 1.06. Transects designed to run perpendicular and parallel to the coastline to cover ecological gradients and areas where high and low variability were expected. Video classification involved identifying primary and secondary substrates and benthic biota, as well as biota density. Primary substrate was classified as sand, rhodoliths (hard structures of coralline algae typically at low points on sandy substrates), obscured reef (hard substrate covered with sand veneer), flat reef, or low, medium, or high profile reef. Biota categories were *Ecklonia spp.* (kelp), other macroalgae and sessile invertebrates.

### Predictive modelling of habitats

Bathymetry and derived terrain datasets were used as input to species distribution models for predicting substrate type and biota at unsampled locations. Classification trees (CTs) were developed in S Plus ® 8.2 for Windows (TIBCO Software Inc, Palo Alto, California, USA) and used to predict (in order) substrates, benthic biota, and finally lobster distributions.CTs explain the variation of a response variable by one or more predictor variables and are constructed by recursively partitioning data and splitting into mutually exclusive groups. The objective is to partition the response into homogeneous groups while keeping the tree size small. Splitting continues until the stopping criterion (e.g. minimum deviance) is reached then the tree is pruned back to an optimal size using cross-validation. The benthos datasets from video observations were randomly split using the set seed function in S Plus before modelling, with 75% of the data used to develop models while 25% was held back as a validation dataset to test the models predictive ability. The set seed function puts the random number generator, which is based upon a single uniform random number generator, in a reproducible state so that identical results from multiple runs can be obtained. After full models were developed, a 10-fold cross-validation was used to identify key predictors and optimal tree size based on the misclassification rate threshold of 15% [20]. The amount of variance in substrate and biotic distribution explained by the models was calculated using adjusted deviance (Adj D^2^). This measure was chosen as it takes into account the differences in the number of observations and parameters used in the development of each model [19].

Separate models were developed for each type of substrate. Video observations of medium and high relief reef were merged together with low relief reef creating a comprehensive reef class. Classes modelled to create a full coverage substrate map were sand, rhodoliths, obscured reef, flat reef and reef. Full models for biota categories were developed using bathymetry, terrain datasets and substrate types.

### Predictive modelling of lobsters

Lobster presence and absence data were derived via catch data from three hundred and thirty (330) standard commercial-sized, baited pots (described above). It is common for species distribution models (SDMs) to use presence/absence data to predict the distribution of a species as a first step to understanding species presence and absence in a landscape (e.g. in this case, seafloor habitats). In addition, frequency histograms of lobsters per pot show a Poisson distribution, with most pots having either one or no lobsters (Figure S1). For this study, catch data were combined across years and any pots with lobsters were treated as present. The Classification tree (CTs) for the lobster model was developed using 75% (chosen using the set seed function in S Plus) of the pot catch data over three years as the dependent variable with predictor variables from bathymetry, terrain, substrate and benthic biota classes. Like substrate and biota models, the 10-fold cross-validation method was used to determine key predictors and optimal tree size. The remaining 25% of the data were used to validate model.

### Model evaluation

Model accuracy was assessed using receiver-operator characteristics (ROC) analysis (ROC AUC software package, Schröder 2004) which evaluated CT performance from the validation dataset (25% of original dataset). ROC analysis is graphical plot of the sensitivity (true positive rate) and specificity (false positive rate) of a model output, commonly referred to as ROC curves. The larger the ‘area-under-the-curve’ (AUC), the more effective the model at prediction. P_fair_ was chosen as the threshold to convert predicted probabilities of occurrence to presence/absences values, by balancing the number of false positives and false negative predictions. It also provides a measure of how well the model predicts both presences and absences.

Full coverage maps of the Leeman site were created once the final models were applied to the predictor datasets. The first map produced for each category modelled showed the probability of presences at 3 m pixel resolution. Binary presence/absence maps were then constructed using the P-fair probability thresholds from ROC analysis.

### Spatial dependence

Species distribution data often display spatial dependence where locations close together exhibit more similar values than those further apart. This is due to dependence of a response variable on explanatory variables (e.g. physical structure) that are themselves spatially structured [29], [30]. One of the key assumptions of statistical analyses is that sample data are independent and violation of this assumption may bias parameter estimates and inflate accuracy [30]. Therefore, it is necessary to understand the effect spatial dependence has on species distribution models. Patterns of spatial dependence of substrate and biota categories were analysed using indicator semivariograms (WinGsLib 1.5.6, Statios Software and Services). In the presence of spatial autocorrelation, the amount of deviance it explained in each model was calculated. Then, the effect on model accuracy was estimated by testing models against blind validation data that was spatially independent from the model data.

## Results

### Benthos observations

A total of 3122 georeferenced video frames were described (Table 2). The ratio between hard and soft substrates observed was approximately 1∶1, with less than 10% of the substrates described being over 1 m in height (medium and high profile reef). The most prevalent primary substrate observed was Rhodolith beds (31%). Macroalgal dominant communities (not including *Ecklonia*) accounted for 29% of frames observed and included mostly red algae. Sessile invertebrate (mostly undifferentiated sponges) dominant habitats accounted for 20% of frames observed while *Ecklonia* dominant communities accounted for 16%. A total of 2087 lobsters (*P. cygnus*) were caught in pots over the three year survey.

**Table 2:** Occurrence of substrate and biota categories observed in frames from towed video footage.

Category	Number of frames	% frames observed
Total video frames classified	3122	100
Substate		
Sand	442	14
Rhodolith	992	31
High Reef	55	2
Medium Reef	147	5
Low Reef	478	15
Flat Reef	620	19
Obscured Reef	412	13
Biota		
*Ecklonia* (kelp)	536	16
Other Macroalgae (mixed)	906	29
Sessile invertebrates	636	20
Hard coral	1	<1

### Model fit and variable contribution

Substrate models explained 53% to 87% of the total deviance (AdjD^2^) (Table 3) indicating solid associations with geophysical environmental variables. CT models developed for hard substrates had the greatest associations, with reef models explaining 73% (low reef) to 86% (flat reef) of deviance. Rhodoliths also showed strong associations with environmental variables, with the model explaining 81% of deviance. In contrast, models for soft substrates including sand and obscured reef (hard substrate with sandy veneer) explained less than 70% of the total deviance (Table 3). Bathymetry and detrended bathymetry were the most influential variable for most substrate models, accounting for 18 to 83% of explained deviance (see Table 4). Focal variables (standard deviation or mean surface area around a focal cell) were also influential, with standard deviation of 7 m kernel radius from focal cell (F7s) retained in four of the six substrate models (Table 4). Dropping this term reduced deviance explained by up to 48% for reef. Dropping the focal variable that calculates the mean surface area within 21 m from the focal cell (F21M) from obscured reef, rhodoliths and sand reduced deviance explained by 79%, 17% and 6% respectively. Other geophysical variables generally accounted for less than 10% of explained deviance.

**Table 3:** Predictive performance of substrate, biota and lobster models was evaluated using the area-under-the-curve of receiver-operating characteristics (ROC) to determine the discriminatory ability of classification tree models.

Validation data (25% of dataset withheld from model development)								Validation data (Spatially independent)				
	Area under curve (Bootstrap)	Threshold for presence	% Sensitivity	% Specificity	% Correct	Adjusted D^2^	# Terminal nodes	Area under curve (Bootstrap)	% Sensitivity	% Specificity	% Correct	Adjusted D^2^
Flat Reef	0.61	0.22	34	85	75	86	13	0.61	33	85	73	83
	(0.56–0.65)							(0.54–0.67)				
Low Reef	0.76	0.20	63	78	75	73	8	0.74	60	78	74	72
	(0.71–0.79)							(0.71–0.77)				
Obscured Reef	0.72	0.19	48	94	88	53	6	0.72	48	91	86	53
	(0.67–0.78)							(0.67–0.77)				
Reef	0.77	0.11	70	76	75	83	9	0.74	70	74	79	82
	(0.73–0.81)							(0.70–0.78)				
Sand	0.74	0.13	76	65	66	68	8	0.71	71	61	61	62
	(0.69–0.78)							(0.67–0.75)				
Rhodoliths	0.77	0.23	66	78	74	81	43	0.75	63	78	73	79
	(0.71–0.85)							(0.70–0.81)				
*Ecklonia* (Kelp)	0.94	0.15	90	84	85	48	7	0.82	78	74	76	39
	(0.89–0.97)							(0.77–0.87)				
Other macroalgae	0.70	0.22	60	68	66	70	35	0.68	60	68	66	70
	(0.66–0.74)							(0.64–0.72)				
Sessile inverts	0.80	0.21	60	87	81	87	12	0.75	60	87	81	87
	(0.77–0.84)							(0.71–0.79)				
*Panulirus cygnus*	0.88	0.46	90	67	80	64	8	0.79	81	61	73	57
	(0.79–0.94)							(0.72–0.86)				

Correct classification, sensitivity and specificity results were used to evaluate prediction accuracy, using P_fair_ as the threshold. Validation data consisted of 25% of dataset withheld from model development. The effect of spatial dependency on model accuracy was investigated using validation data that was spatially independent.

**Table 4:** The contribution of predictor datasets for the substrate models as percentage of explained deviance.

Variable	Code	Sand	Rhodoliths	High reef	Medium reef	Low reef	Flatreef	Obscured reef	Reef(H+M+L)
Bathymetry (depth)	DTH		13	14		12		15	18
Bathymetry (detrend)	DETRND	83	25		8		43	6	
Slope	SLP	<1			9				
Aspect	ASP	<1	9		<1		<1		
Rugosity (surface area)	SURFA								6
Curvature	CURV								4
Profile curvature	PROFCURV								
Plan curvature	PLANCURV		1	11		3			
Focal analysis (surface area, mean, 21 m radius)	F21M	6	17		17			79	
Focal analysis (surface area, stdev, 21 m radius)	F21S					3	41		3
Focal analysis (surface area, stdev, 7 m radius)	F7S		6	75	45	45	<1		48
St Deviation (12.5 m radius)	STD5		7						16
St Deviation (25 m radius)	STD10				3				
St Deviation (62.5 m radius)	STD25		2				13		
Range (12.5 m radius)	RNG5								
Range (25 m radius)	RNG10	9			5				
Range (62.5 m radius)	RNG25	2	2		8				
Hypsometric index (12.5 m radius)	HYP5		2						5
Hypsometric index (25 m radius)	HYP10					4			
Hypsometric index (62.5 m radius)	HYP25		16		2	30			

For biota categories, sessile invertebrates had the highest deviance explained by the model with an AdjD^2^ of 87%, followed by other macroalgae (70%) and *Ecklonia*(48%). *Ecklonia* and sessile invertebrates showed strong associations with depth contributing 57% and 45% of explained deviance respectively, followed by hard substrate types which contributed to 17% and 41% of explained deviance, respectively (Table 5). In contrast, the contribution of variables for other macroalgae was spread more evenly among predictors, with range (62.5 m radius) contributing the most explained deviance at 24%.

**Table 5:** The contribution of predictor datasets for the biota models as percentage of explained deviance.

Variable	Code	*Ecklonia*	Inverts	Other Algae
Bathymetry	DTH	57	45	8
Bathymetry (detrend)	DETRND		<1	9
Slope	SLP			<1
Aspect	ASP			2
Rugosity	SURFA			1
Curvature	CURV			
Profile curvature	PROFCURV			
Plan curvature	PLANCURV			
Focal analysis (surface area, mean, 21 m radius)	F21M	13		13
Focal analysis (surface area, stdev, 21 m radius)	F21S		<1	
Focal analysis (surface area, stdev, 7 m radius)	F7S		<1	
St Deviation (12.5 m radius)	STD5			
St Deviation (25 m radius)	STD10			
St Deviation (62.5 m radius)	STD25			<1
Range (12.5 m radius)	RNG5			4
Range (25 m radius)	RNG10	12		
Range (62.5 m radius)	RNG25		<1	24
Hypsometric index (12.5 m radius)	HYP5			2
Hypsometric index (25 m radius)	HYP10			
Hypsometric index (62.5 m radius)	HYP25			
Reef		17	23	10
Flat Reef			18	
Obscured Reef				8
Sand				18
Rhodoliths				

**Figure 2:**
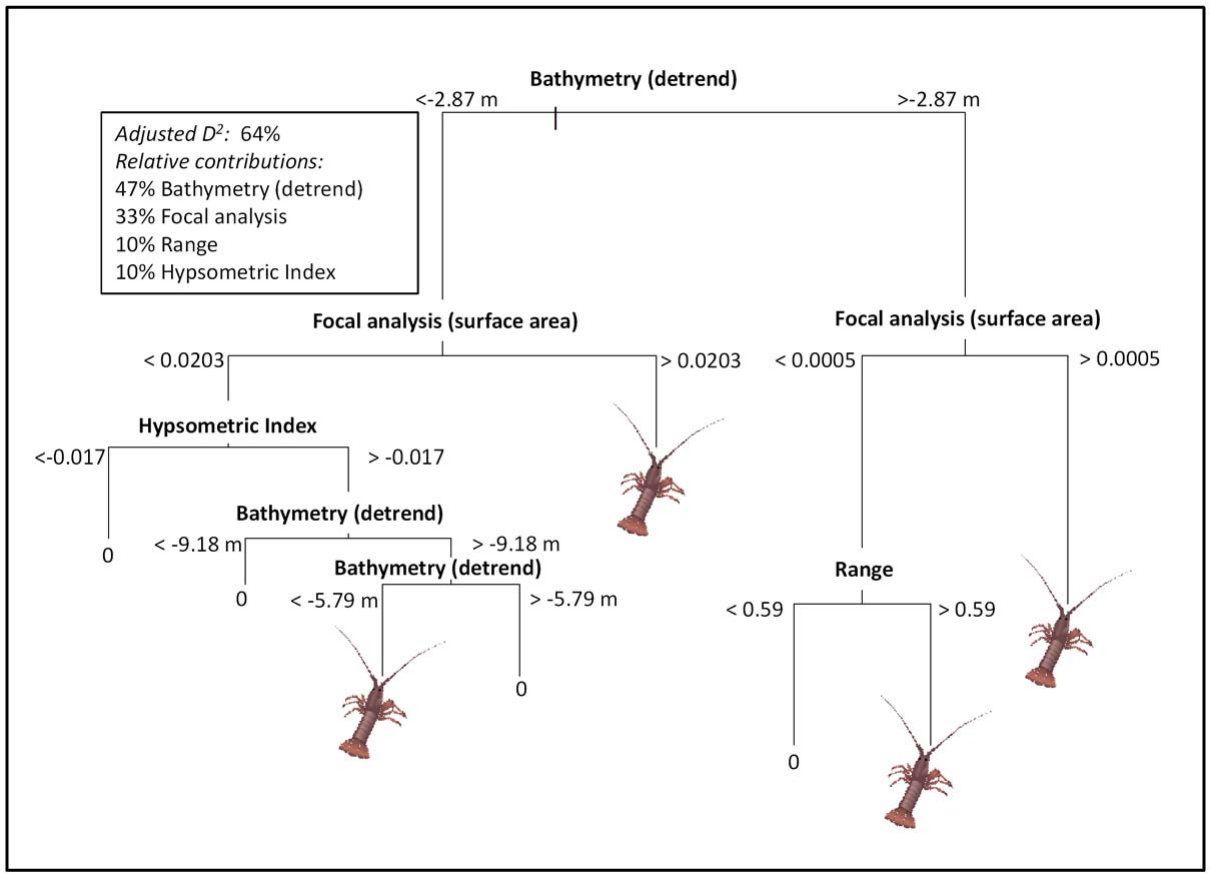
Final classification tree model for the presence/absence of western rock lobster, *Panulius cygnus*. Focal analysis was calculated based on standard deviation of surface area over a 7 m kernel radius. Hypsometric Index and Range were calculated over a 12.5 m and 62.5 m kernal radius, respectively. Bathymetry (detrend) was the most influential variable, contributing to 47% of the variation explained, followed by focal analysis (33%), range (10%) and hypsometric index (10%).

The CT model for lobsters explained 64% of total deviance. The model retained only geophysical variables, including three based on local neighbourhood measures; hypsometric index with 12.5 m kernel radius (HYP 5), range with 62.5 m kernel radius (RNG 25) and focal analysis using standard deviation statistic (F7s), as well as depth (Fig. 2). Depth and focal analysis contributed to 80% of explained deviance while the other variables each contributed 10% of explained deviance.

### Model evaluation

Of the six substrate models, five had acceptable predictive power (AUC range between 0.72 and 0.77, Table 3). Flat reef had poor predictive power with an AUC value of 0.61, despite the model explaining the highest total deviance. The ability of the model to correctly predict presence of flat reef was low, as suggested by low sensitivity (34%). Sensitivity values of other substrates ranged from 48 to 76% (Table 3). For all substrates except sand, specificity values (correct absences) where higher than sensitivity values and ranged from 65 to 94% (Table 3). The total number correct predictions (both presence and absence) ranged from 66% for sand to 88% for obscured reef (Table 3).

Evaluation of *Ecklonia* and sessile invertebrate models showed good predictive power (AUC=0.94 and 0.80, respectively) and correct classification rates above 80%. *Ecklonia* had the best predictive performance with both high sensitivity (90%) and specificity (84%). Other macroalgae model performance was poorer (AUC=0.70), with low sensitivity and specificity values, and correct classification rate (Table 3). For the lobster model, high sensitivity increased overall performance with an AUC value of 0.88 and a correct classification rate of 80%.

In most instances, the effect of spatial dependence on deviance explained by the model or model accuracy was minimal (less than 5%) (Table 3). Deviance explained by the *Ecklonia* and lobster models was reduced by 9% and 10% respectively. However, model accuracy was good according to accepted statistical tests, with AUC values above 0.79 and correct classifications above 73%.

### Mapping

**Figure 3:**
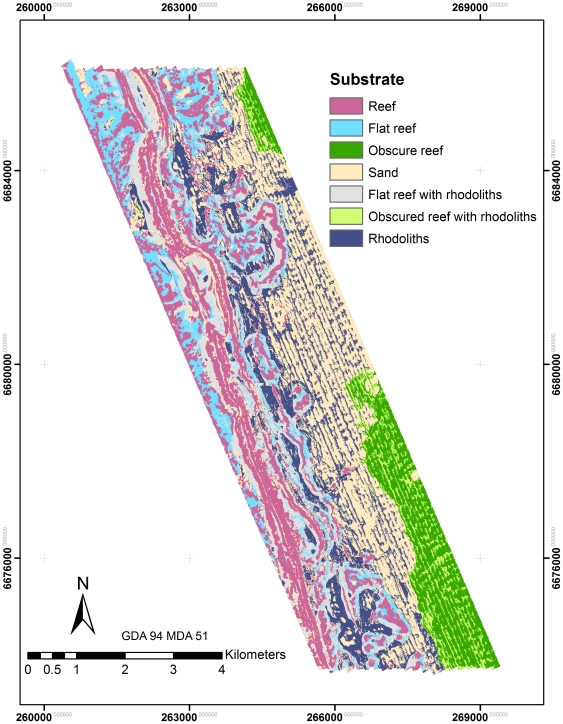
Map of integrated substrate distribution used to help predict lobster distribution.

**Figure 4:**
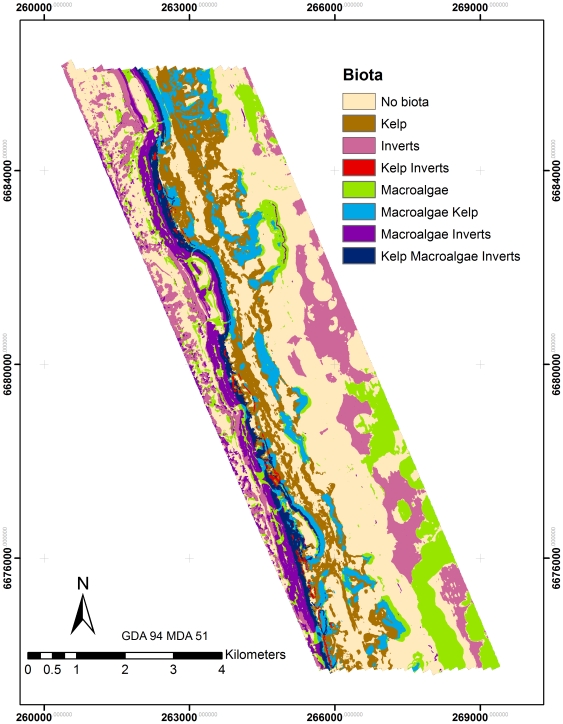
Map of integrated biota distribution used to help predict lobster distribution.

**Figure 5:**
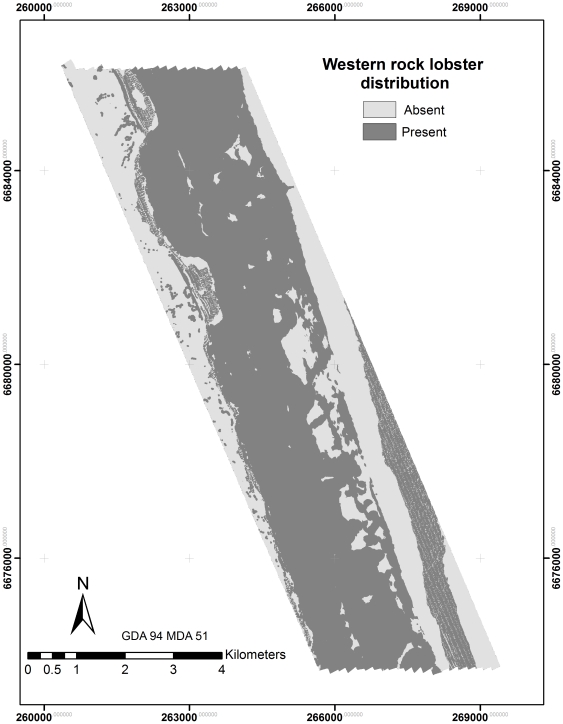
Map of western rock lobster distribution.

Final maps provide a detailed representation of dominant substrates and biota in the study area (Fig. 3, Fig. 4 and Fig. 5). The maps document a clear gradient of *Ecklonia* dominance on the ridge and lee of the reef that aligns with lobster distribution. Sessile invertebrates dominate the flat reef structure in deeper waters (>65 m) and sandy substrates in shallow waters. Rhodoliths tend to dominate on the shallow side of the reef and extend into the sandy substrates to the east. Some mapping artefacts are visible in areas of soft substrates, where boat motion likely introduced striping effects.

## Discussion

The development of spatially explicit, detailed habitat maps in this study has allowed us to accurately represent the benthic environment in an area of known importance for the western rock lobster. The models can be used to explore complex geomorphic characteristics and the major drivers in benthos distributions in deep water lobster habitats. For three of the four biota categories (including lobster), more than 60% of the variation in distribution could be explained by depth and geophysical variables. This confirms that depth and geomorphology are the principle drivers of biota distribution at this location. Moreover, these variables are ecologically relevant as they reflect important physiological (e.g., light requirements), environmental or ecological (e.g., hard substrates to attach holdfasts) limitations [26], [31].

Though time consuming and expensive to produce, habitat maps are critical for assessing the spatial relationship of important marine resources to their environment. For example, habitat suitability maps for European lobsters showed that they are more abundant on the lee side of peaks or ridges [12], where it has been suggested that they feed most actively. Similarly, the western rock lobster also appears to have a preference for the leeward side of the reef ridge (Fig. 3 and Fig. 5). Based on our current understanding of lobster ecology, it was expected that substrate types such as boulders [32], coral reef [33] or rocky reefs with mixed kelp assemblages [18], [34], could be significant variables in defining lobster distribution. However, our model for lobsters did not incorporate any of the substrate or biota classes developed from towed video surveys.

Integrating geo-referenced pots, hydroacoustic surveys and detailed habitat maps has been demonstrated as an effective and efficient method in a number of lobster studies [35]–[37]. In this study, the combined benefits of pots and seafloor mapping have revealed significant and somewhat unexpected effects of habitat on lobster distribution. We found that geophysical variables based on local neighbourhood analysis were key predictors of lobster distribution, suggesting that a fine scale mix of terrain is important to western rock lobster. The selection of geophysical variables indicates a preference for geomorphologic complexity. Studies on different lobster species around the world have also shown a strong association between sea floor complexity and lobster distribution [12], [38]–[40]. Habitat complexity is mainly used as a proxy for shelter quality, where highly complex habitats provide greater protection from predators.

Complex habitats may also support high abundances of organisms that serve as food for lobsters, as shown in seagrass meadows [41]. On the other hand, food resources are sometimes spatially segregated from refuge habitats [42], [43] and is supported by the strong association between lobster abundance and proximity to edges of habitats that differ in shelter quality and resource availability [12], [43]. Thus, the geophysical variables used in the model in this study (hypsometric index, focal analysis, and range) may typify a landscape with a juxtaposition of habitats suitable for both sheltering and foraging for western rock lobster. Lobster behaviours such as feeding, foraging, social interaction, movement and migration are regulated by a combination of chemosensors, hormones and magnetoreceptors. The inclusion of environmental variables that relate to the sensory biology, such as oceanographic conditions (e.g. wave energy, water currents) will further help to understand the complex relationship of lobsters to the range of habitats they utilise.

An earlier study on the relationship between the western rock lobster and habitat established a significant (though moderate) association, with mixed assemblages dominated by *Ecklonia* sp. accounting for 28% of variation in abundance of western rock lobster [18].While *Ecklonia* and reef are abundant at the study site, we found that a fine scale mix of terrain is the strongest driver of lobster distributions. The approach used in this study provides a more holistic view of the ecological and spatial structure of the western rock lobster and their preferred habitats. Crucially, the relationship between fine scale features in the landscape and lobster distribution will have strong implications for the spatial management of the western rock lobster fishery. The fundamental first step will be assessing how much preferred habitat is available to the western rock lobster within its geographical range.

The difference in model variables in the two western rock lobster studies likely relates to the scale of characterisation of habitats. Bellchambers et al. [18] used a broad-scale presence-absence approach, while our study used bathymetry data (at 3×3 m pixel resolution) to characterise terrain down to 12.5 m kernels (Table 1). The role of scale in habitat characteristics on abundance and distribution has been investigated in the California spiny lobster (*P. interruptus*) [39] and discussed with respect to the American lobster (*Homarus americanus*) [43]. These studies suggest that lobsters respond to habitat characteristics at local (e.g. shelter) and landscape scales (e.g. kelp dominated habitat), with microhabitat influencing the distribution of lobsters within patches of broadly suitable habitat. The results of the present study, in conjunction with Bellchambers et al. [18], suggest similar broad and fine scale habitat characteristics influence the distribution of the western rock lobster.

### Conclusion

The development of spatially-explicit, detailed distribution maps in this study allowed an area of known importance to the western rock lobster to be accurately characterised, despite the depth (60–90 m). We were able to demonstrate for the first time that kelp and reef are not the intrinsic drivers of lobster distribution off the coast of Western Australia, as first thought. Clearly, kelp and reefs do play a role in the ecological structure of western rock lobsters, as they align closely with lobster distribution. The use of species distribution models in this study, however, shows that it is the fine scale features within the geophysical landscape that strongly influence patterns in lobster distribution. Our results imply that the juxtaposition of habitat, terrain and scale are most likely to define the ecological niche of western rock lobster. In essence, the preferred habitat is not limited by the presence of reef or kelp, rather a geomorphic complexity that is likely related to the presence of quality shelter and food resources. This study provides new and critical information to the ecological and spatial structure of western rock lobster which will be an integral part of the way ecosystem –based fisheries management (EBFM) is implemented for the sustainability of the western rock lobster fishery. For example, we can start to predict the carrying capacity of the WRL, based on how much preferred habitat is available within its geographical range. Congruent with this is the chance to assess the potential impacts of fishing without the confounding effects of habitat quality [44]. More importantly, understanding the spatial structure of habitats as perceived by the organism in question is crucial to gaining insight into the ecological processes necessary for population persistence and maintenance, which are fundamental to managing a sustainable fishery. The next step is to determine the most appropriate landscape configuration, composition and scale to refine the ecological niche for western rock lobster. However, understanding the interaction of the organism with their physical habitat must extend past the just sexually mature lobsters (brood stock) and move towards conserving an intact life history and all the habitats associated with different life stages.

## Supporting Information

Figure S1 **Frequency distribution of the number of lobsters caught per pot.**
(DOCX)
