# Supplementary material for: Modelling Deep Water Habitats to Develop a Spatially Explicit, Fine Scale Understanding of the Distribution of the Western Rock Lobster, Panulirus cygnus
Source: PLoS One. 2012 Apr 10;7(4):e34476. doi: 10.1371/journal.pone.0034476 (PMC3323630; doi:10.1371/journal.pone.0034476)
Supplement: Figure S1 — Frequency distribution of the number of lobsters caught per pot. (DOCX) [file pone.0034476.s001.docx]

Supplementary Information:


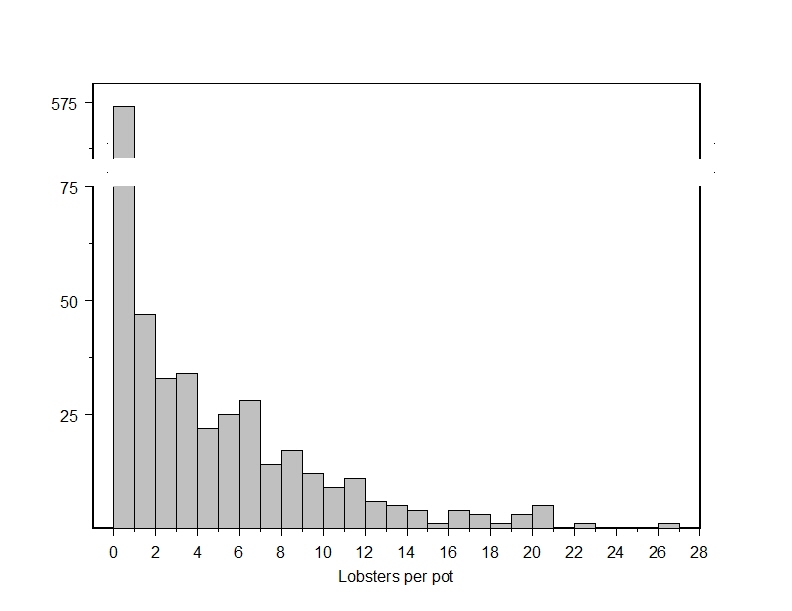


Figure 1: Frequency distribution of the number of lobsters caught per pot.
